# Community-Based Prescribing for Impetigo in Remote Australia: An Opportunity for Antimicrobial Stewardship

**DOI:** 10.3389/fpubh.2017.00158

**Published:** 2017-07-12

**Authors:** Stefanie Jane Oliver, James Cush, Jeanette E. Ward

**Affiliations:** ^1^Pharmacy Department, Western Australia Country Health Service (WACHS)-Kimberley, Broome, WA, Australia; ^2^Paediatrics Department, Western Australia Country Health Service (WACHS)-Kimberley, Broome, WA, Australia; ^3^Kimberley Population Health Unit, Western Australia Country Health Service (WACHS)-Kimberley, Broome, WA, Australia; ^4^Nulungu Research Institute, University of Notre Dame Australia, Broome, WA, Australia

**Keywords:** antimicrobial stewardship, community-based prescribing, remote indigenous health, impetigo, *Streptococcus pyogenes*

## Abstract

**Background:**

To support antibiotic prescribing for both hospital and community-based health professionals working in remote North Western Australia, a multidisciplinary Antimicrobial Stewardship (AMS) Committee was established in 2013. This Committee is usually focused on hospital-based prescribing. A troubling increase in sulfamethoxazole/trimethoprim resistance in *Staphylococcus aureus* antibiograms from 9 to 18% over 1 year prompted a shift in gaze to community prescribing.

**What we did:**

Finding a paucity of relevant research, we first investigated contextual factors influencing local prescribing. We also designed a systematic survey of experts with experience relevant to our setting using a structured response survey (12 questions) to better understand specific AMS risks. Using these findings, recommendations were formulated for the AMS Committee.

**What we learned:**

Prescribing recommendations in a regional *Skin Infections Protocol* had previously been altered in December 2014. From 15 experts, we received 9 comprehensive responses (60%) about AMS risks in community prescribing. If feasible, prescribing audits also would have been valuable. Ten recommendations regarding specific antibiotic recommendations were submitted to the AMS Committee.

**Strengthening AMS in remote settings:**

As AMS Committees in Australia usually focus on hospital-based prescribing, novel methods such as external expert opinion could inform deliberations about community-based prescribing. Our approach meant that this AMS Committee was able to intervene in the 2017 organizational review of the regional *Skin Infections Protocol* used by prescribers likely unaware of AMS risks. This experience demonstrates the value of incorporating AMS principles in community-based prescribing in context of a remote setting.

## Introduction

Antimicrobial stewardship (AMS) is a global response to a universal imperative to promote responsible prescribing (1). Some 700,000 people die of bacterial infections resistant to effective antibiotics every year (2). In Australia, considerable attention has been focused on antibiotic prescribing in hospitals (3). Yet antibiotic use in community settings in Australia is high (4, 5). In 2014, almost half (46%) of Australians had at least one antimicrobial dispensed to them with an overall rate of 23.8 “daily defined doses” per 1,000 inhabitants per day (4). Rates of antibiotic resistance behoove both global and local responses (6).

The Kimberley region of North Western Australia is one of Australia’s most remote. It has a small population of approximately 40,000 people spread over an area as large as Germany. About half of the resident population is Aboriginal (7). Health outcomes are inequitable (7). Delivery of healthcare is challenging. Hospital services for the Kimberley region are provided exclusively by Western Australian Country Health Service (WACHS). While WACHS also provides primary healthcare clinic services, it is not the monopoly provider. Remote Aboriginal communities are served by a mix of WACHS-managed primary healthcare services and Aboriginal Community Controlled Health Organisation (ACCHO).^1^ This plurality of primary healthcare services requires partnership between them (8).

In 2013, WACHS created an AMS Committee for the Kimberley region with membership from all relevant disciplines from both WACHS and ACCHO services. Since then, it has focused on hospital-based prescribing, particularly as tools such as the National Antibiotic Prescribing Survey are readily available to do so.^2^ In December 2015 however, antibiograms received by the AMS Committee showed an increase across the region in methicillin-resistant *Staphylococcus aureus* (MRSA) resistance to sulfamethoxazole/trimethoprim also known as “co-trimoxazole” (SXT) from 9% in one 12-month period (July 2013–June 2014) to 18% in the next (July 2014–June 2015). In contrast, Australian rates of MRSA-resistant to SXT usually range from 2.5 to 11.9% (4). In an AMS Committee dominated by hospital members and usually dealing with hospital prescribing, this troubling increase in SXT resistant MRSA prompted an urgent pivot toward community-based prescribing.

## What Did We Do to Enhance Our Understanding of Prescribing Risks and Benefits?

To assist a hospital-focused AMS Committee better understand community-based prescribing, a working group (SO, JC, JW) was tasked with summarizing evidence and risks of prescribing choices when treating bacterial skin conditions affecting Aboriginal people in remote community clinics. No additional resources were available.

### Identification of Factors Influencing Prescribers

We first informally examined sources of information, regulations, and experiences likely influencing prescribers. Skin infections including impetigo are very common among Aboriginal people supplied by government with housing inadequate for cultural demands and social use. From 10 population prevalence studies reporting data for children living in remote Aboriginal communities of northern Australia, the median prevalence of impetigo reported from these studies was 44.5% (9). Furthermore, the organism predominantly driving impetigo is Group A *Streptococcus pyogenes* (GAS) rather than *Staphylococcus aureus* (10). As a result of this epidemiological picture, the aims of prescribing to treat impetigo in the Kimberley are twofold: first, to accelerate skin healing and, also, to prevent serious GAS sequelae such as acute rheumatic fever and acute post-streptococcal glomerulonephritis (APSGN).

All authorized prescribers in Australia can access national Therapeutic Guidelines online.^3^ Because of the plurality of service organizations in our region as previously described, a suite of about 20 Kimberley-specific clinical protocols also is promoted upon arrival in the region at staff orientation and during in-service.^4^ These protocols are designed to support inexperienced health professionals unused to working in a setting as remote as the Kimberley and also to minimize prescribing deviations outside the agreed Kimberley standard drug list (KSDL) common to all services. We looked closely at the prescribing sections of one of these protocols, namely the *Kimberley Skin Infections Protocol*.^5^

We sought relevant pharmacological literature, also searching widely for community-based prescribing research conducted in remote settings with Aboriginal people rather than mainstream urban settings. We investigated the feasibility of a prescribing audit in the Kimberley but found both logistics and costs prohibitive as no standard audit tools were available. In addition, we resolved to obtain expert clinical input.

### Expert Clinical Input

**Figure 1:**
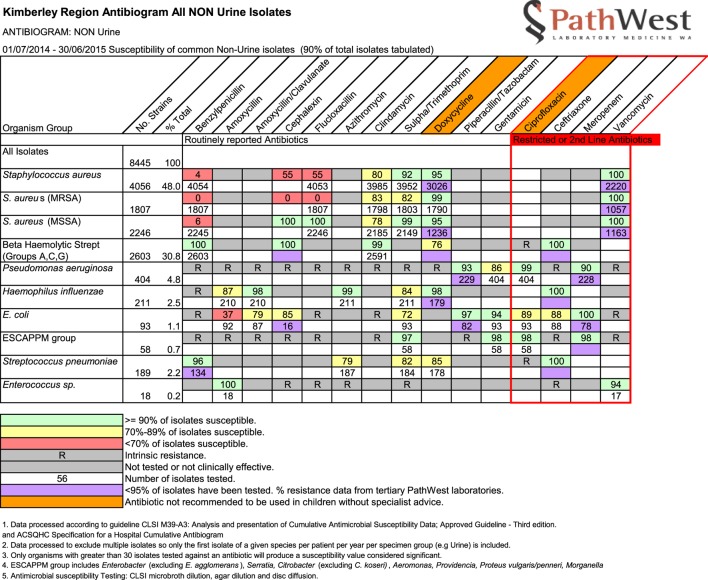
Example of antibiogram discussed by antimicrobial stewardship Committee in December 2015.

The AMS Committee agreed we could approach independent experts and reviewed the names of proposed experts for disciplinary background and relevant experience working in remote Aboriginal healthcare. Each of these then was first approached by email by SO requesting assistance in this AMS project. Our invitation outlined the purpose of our request and provided six article abstracts found in our literature review (available upon request from SO). We also attached the Kimberley antibiograms for the period July 2014–June 2015 (Figure 1) with the link to the current *Kimberley Skin Infections Protocol*. A structured response sheet presenting a list of 12 questions with a deadline for completion was used to guide expert review (Box 1). Development of these specific questions came out of our initial examination, earlier informal consultations, and literature review. About 4 weeks after initial invitation, one of us (JC) made a prompting telephone call or collegial reminder to any non-respondent to encourage response.

Box 1 **Twelve questions asked of experts in a structured response survey.**

- Is prescribing according to the Protocol driving up multi-methicillin-resistant *Staphylococcus aureus* (MRSA) rates?
- Does the option of 3-day duration of co-trimoxazole versus 5-day duration raise risk of increasing multi-MRSA rates?
- What is the best use of co-trimoxazole in community prescribing in a region with very high rates of cMRSA, and where it remains our most useful oral agent for cMRSA?
- Does co-trimoxazole bring corroborated benefit in reducing initial or recurrent acute rheumatic fever as does LAB?
- Should the first-line advice given in the Protocol be changed to strongly emphasize LAB as first line and an oral alternative as second line in very limited situations?
- What is the role of oral penicillins and/or cephalexin as first-line prescribing options?
- Cephalexin is prescribed as a first-line antibiotic for impetigo in non-Aboriginal patients. Should it be specified as a first-line antibiotic for community-based treatment of impetigo in the Kimberley?
- Should a revision of the protocol specify a focus on Aboriginal children 0–17 years of age rather than, as currently, no age specified?
- Should there be a regular census (audit) conducted periodically in the Kimberley by which a large number of swabs were taken simultaneously according to a census protocol to provide useful antiobiograms and other AMS-related data, or can we rely on our current antiobiograms to inform decisions?
- Is there a need to design and put in place a long-term monitoring system for antibiotic resistance in the Kimberley? What might be a warning light that we need to protect co-trimoxazole for use in cMRSA?
- Should a standardized concordance audit be designed to ask each clinic or PHC service to show how a patient with a skin infection was treated and whether according to the protocol using a standardized tool?
- Should the revised protocol alert clinicians to multiple previous presentations for skin infections as reason to refer to Environmental Health services using EH referral form?

### Progress Reporting to AMS Committee

Throughout the process, we prepared project updates either verbally at meetings or in writing to the AMS Committee. Suggestions we submitted regarding process, selection of experts, and findings.

## What Did We Learn?

### Guidelines and Other Sources of Prescribing Directions in the Kimberley

Prescribers in the Kimberley are either medical practitioners or, in circumscribed clinics in accordance with the WA Medicines and Poisons Act (2014) and regularly updated legislation, credentialed Remote Area Nurses (RANs). A pre-existing version of the *Kimberley Skin Infections Protocol* produced originally in 2010 had flucloxacillin orally for 6 days or, if adherence to an oral schedule would be difficult, intramuscular injection of long-acting benzathine benzylpenicillin (LA Bicillin^®^) (LAB) for both adults and children.

This 2010 *Protocol* had been revised in some haste during 2014 in the context of an outbreak of APSGN in the Kimberley on the basis that GAS impetigo was the causative trigger. Changes introduced during this revision had included a prescribing recommendation to use SXT as an equivalent first-line oral alternative to intramuscular LAB. The AMS Committee itself had no formal record of these changes or AMS scrutiny. Released in December 2014, this version of the *Protocol* had been in place for about 1 year at the time that the AMS Committee first received data showing increasing MRSA resistance to SXT across the region. Anecdotally through emails between prescribers and queries to pharmacists, the use of SXT as first-line oral antibiotic for impetigo had increased over that year. A system of Standing Orders authorized by the Director-General of WA Health listed SXT as well as intramuscular LAB for use by RANs to treat “skin infections.”

None of the clinical record systems used in primary healthcare in the Kimberley could generate prescribing data linked to impetigo treatment in order to quantify actual prescribing behavior as part of this investigation. Examining turnover of stock and trends in SXT ordering by interrogating pharmacy imprests of each remote clinic setting also was precluded on the basis of available resources and, in any case, would have been far less precise.

### Expert Input Adds to Published Evidence

From 15 experts and clinicians asked to provide input, we received 9 completed response sheets (60%) providing an appropriate diversity of disciplines including general practice, pediatric infectious diseases, clinical microbiology, clinical pharmacy, and experienced remote area pediatrics. Although every article provided with our structured response survey had been utilized by at least one respondent completing this section, crucially one had been used by all (11). As one additional article was cited by one respondent only (12), we were reassured that the resources we had provided were comprehensive and sufficient for respondents.

One of us, JW, was charged with the task of de-identifying and collating written responses. Extensive comments were considered over dedicated meetings of the working group. This process affirmed that antibiotic exposure raises risk for antibiotic resistance at individual as well as community and regional levels. All expert survey respondents confirmed the AMS risks and trade-offs inherent in first-line antibiotic choices in treating Aboriginal children with impetigo. These also included the options of oral versus intramuscular routes. A direct correlation between increasing antibiotic resistance, as observed by the locally produced antibiograms, and increasing SXT antibiotic exposure could not be excluded. Survey responses focused attention on a single study conducted in the Northern Territory comparing an oral antibiotic alternative to intramuscular LAB for the first-line treatment of impetigo in Aboriginal children (11). As explained by the researchers, their selection of SXT as this oral antibiotic alternative would cover MRSA as a causative organism and their previous *in vitro* study demonstrating susceptibility of GAS to SXT. They also explained that, if *S. aureus* was causative of impetigo in any given individual, then intramuscular LAB would be insufficient (11). Survey respondents’ comments generally agreed that impetigo within Australian Aboriginal populations is driven by *S. pyogenes* (GAS). Regardless of concomitant *S. aureus* carriage in skin infection, treatment of *S. pyogenes* alone results in clinical resolution. In the Kimberley, clinicians must manage high rates of GAS infections but also a wide range of clinical manifestations of *S. aureus* infections, ranging from simple boils and cellulitis to complex deep tissue and joint infections and life threatening sepsis. Our rates of community-acquired methicillin-resistant *Staphylococcus aureus* (ca-MRSA) are high. SXT should remain one of the region’s most precious oral antibiotic agents for ca-MRSA infections demonstrated to be sensitive to SXT.

Responses from our review also suggested the importance of separately addressing community-based treatment of *S. aureus* infections in any revision of the *Kimberley Skin Infections Protocol*. Treatment of non-impetigo cases known or suspected to be caused by *S. aureus* needed greater detail. As decided by the AMS Committee, a separate guideline for inpatient management will also be produced with clear links from the community-based Protocol to a hospital-based prescribing protocol. This hospital protocol for more severe infections requiring admission is under-way and will be informed by a concurrent study of diagnosis, treatment, and prescribing of impetigo in Aboriginal children once hospitalized (13).

Expert responses also helped us as a working group to highlight specific methodological aspects of previous research. One study showing a reduction by day seven of *S. aureus* carriage in Aboriginal children with impetigo had compared one of two previously un-trialed SXT regimes against intramuscular LAB based on non-adherence concerns (11). Specifically, the trial compared a 3-day course of SXT at a standard dose twice daily versus a 5-day course at a novel double-dose once-daily but, importantly, all doses were “directly observed” (11). Also known as directly observed therapy (DOT), such a standard for clinical treatment of patients with impetigo in the Kimberley was discussed at length by the working group. In addition, this trial had not assessed SXT resistance. Methods including pharmacokinetic/pharmacodynamic (PK/PD) analyses to determine PK/PD indices to optimize efficacy and avoid emerging resistance are needed for SXT (14, 15). Furthermore, it is known that SXT has a shorter half-life in children (16). As a working group so advised, we concluded that all antibiotic dosing regimes should be conventional and, in keeping with AMS principles and the evidence before us, conservative in a remote setting. Indeed, we were advised that the eTG specifies cephalexin for impetigo in non-Aboriginal children.

Survey responses also reinforced the role of environmental determinants of skin infections in Aboriginal children. As one respondent suggested: *“*… *we have to make a start in addressing the environmental factors that are contributing to high rates of skin infection. It does need to be part of a wider strategy addressing the social determinants of health such as overcrowding though I accept that this is likely to be beyond the scope of the review.”* Our working group agreed.

Readers can request from SO a full copy of the responses with AMS Committee permission.

### Actions by the AMS Committee

Having discussed every written response and common themes, we prepared written recommendations for the AMS Committee in two sections: one, longer and comprising eight recommendations, addressed the first-line oral antibiotic alternative to intramuscular LAB for impetigo in Aboriginal children and, the second, comprising two recommendations about non-impetigo skin infections (Box 2). In response to local contextual factors influencing prescribing, we recommended retaining the option for an empiric oral antibiotic alternative alongside intramuscular LAB for the initial treatment of GAS impetigo in Aboriginal children in remote Australia. Consistent with AMS principles, SXT should be removed as this first-line oral alternative due to a disturbing increase in MRSA resistance to SXT in the region. In a setting of high (and rising) MRSA resistance to SXT, we were confident that we could recommend that SXT should be used selectively especially when other options were readily reaffirmed by experts participating in our survey. Until more evidence is available, we recommended compliance with the Australian Therapeutic Guidelines (eTG) prescribing recommendation for impetigo of cephalexin. Recommendations were also made about audit, community engagement, and environmental determinants (Box 2).

Box 2 **Prescribing recommendations for community-based treatment of impetigo in remote Australia.**

**First-Line ORAL Antibiotic Alternative to Benzathine Penicillin (LA Bicillin) Depot Injection for Impetigo Treatment**

- We recommend keeping the choice of an oral antibiotic alternative to LA Bicillin for the initial treatment of impetigo.
- We recommend, as informed by local antibiograms, that this oral alternative should no longer be sulfamethoxazole/trimethoprim (“co-trimoxazole”) (SXT) due to the concern of increasing methicillin-resistant *Staphylococcus aureus* (MRSA) resistance to SXT in the region.
- We recommend instead that the oral antibiotic alternative, knowing that impetigo is almost always driven by *Streptococcus pyogenes*, be a more narrow spectrum antibiotic. Due to the current evidence base about oral penicillins, we recommend compliance with the current Australian Therapeutic Guidelines recommendations for impetigo in non-indigenous children of cephalexin until more evidence is available.
- Where both penicillin and sulfur allergy are present, we recommend a review of the current suggested alternative of roxithromycin by the maternal and child health sub-committee. Where only penicillin allergy is present, we recommend continuation of the current recommendation in the Protocol, i.e., SXT, noting issues raised about SXT dosage and antibiotic resistance (i.e., twice daily versus once-daily regimes).
- We recommend the need for thorough dialog with parents/families about these choices for impetigo treatment so they are better informed about the options, and are actively involved in the choice of either injection or oral therapy.
- We recommend staff education in ways to administer LA Bicillin in a patient friendly manner and in ways shown to significantly reduce pain in recipients.
- We recommend no change to the advice in the guideline about the routine use of directly observed therapy (DOT) to improve oral antibiotic adherence, i.e., there is no need to introduce DOT as an element of prescribing.
- We recommend impeccable follow up with all patients with impetigo to assess success of antibiotic therapy.

**Non-Impetigo Skin Infections**

1. We recommend that the skin infection protocol continue to be inclusive of all skin infections, however, we recommend far greater clarity for clinicians regarding the clinical assessment to distinguish infection driven by *Streptococcus* (impetigo) versus infection driven by *Staphylococcus* (boils, abscess) versus infections such as cellulitis which are likely a combination. This is critical as treatment and potential complications are different. Currently, the Protocol implies a blanket LA Bicillin injection or oral co-trimoxazole for all presentations. If the patient has a staph driven infection, LA Bicillin is inappropriate.
2. We recommend routine swabbing of any lesion(s) suspected to be *Staphylococcus* driven. Even if MRSA is found to be colonizing the wound, other bacteria may still be driving the infection and need treatment especially in more serious infections.

On the basis of this process and its own deliberations based on the diverse sources of input obtained, the AMS Committee endorsed these recommendations in October 2016 and requested a comprehensive review of the *Kimberley Skin Infections Protocol*. It provided a comprehensive report to the KAHPF Maternal and Child Health SubCommittee responsible for this review (due mid-2017) including an explanation of AMS principles. Additional consultation will take place. As the most recent antibiogram received by the AMS Committee from January 2015 to December 2015 showed continuing MRSA resistance to SXT in the Kimberley (19%), this initiative to apply sound AMS principles to community-based prescribing was timely. Unpublished data also made available shows that the WA121 strain of *S. aureus* now accounts for half of all strains of *S. aureus* found in the Kimberley. WA121 is routinely resistant to SXT (17, 18).

## Strengthening AMS in Remote Settings

This experience identifies future directions for AMS Committees such as ours in remote settings that oversight both hospital-based and community-based prescribing. By obtaining external expert opinion where required in conjunction with relevant literature, this AMS Committee has initiated an organizational review of a regional *Skin Infections Protocol* that is used as a resource by prescribers likely unaware of AMS risks.

There is potential for still more strategies to strengthen AMS in community-based antimicrobial choices such as the case study described here. Although no methodical audits could be undertaken on this occasion to quantify prescribing practices in the Kimberley as part of our project, audits with feedback can provide powerful signals that change prescribing behavior (19, 20). We are hopeful that there will be committed support for such AMS activities in the future including audits and routine reporting to the AMS Committee of drug ordering by regional pharmacies. A survey of medical and nursing prescribers would also have shone light on their perspectives. PathWest has been encouraged to consider inter-regional comparisons and time-series trend analyses of SXT resistance. Certainly, clinical prescribing audits will be needed to monitor the impact of future Protocols.

Culturally informed initiatives could better promote antibiotic adherence in our context, especially when combined with resources proven to support decisions by Aboriginal people for complex diseases in disadvantaged circumstances. Culturally specific resources to explain antibiotics or antibiotic resistance are rare in Australia (21) and non-existent in any Aboriginal language of the Kimberley. As concerns with pain may be acting as an impediment to intramuscular LAB, staff education should also be strengthened. Unfortunately, there are few resources available to upskill clinicians to reduce pain from LAB as an intramuscular injection. Rheumatic heart disease (RHD) Australia has a module within a series on RHD that requires registration to complete. In addition to a project in New Zealand that trialed the use of lignocaine injection and a small vibrating bee-shaped device with ice-pack wings placed specifically in relation to the injection site and nerve fibers (22), an Australian pediatric service based in Townsville serving remote communities through outpatient visits has been trialing a child friendly method, with anecdotally reported success.^6^ Others concur with the need for more patient-centered approaches to penicillin use (23). Specifically, 10 clinical experts in RHD identified characteristics of benzathine penicillin G formulations which could be changed to improve adherence with secondary prophylaxis. Those overwhelmingly put forward included dose interval, pain, and administration mechanism (23). Looking further to the emerging ethical complexities of managing antimicrobial risks for entire populations alongside individual autonomy (24), we suggest that presenting communities with data about antibiotic resistance and entering into genuine long-term discussion about these complexities will better support strategies that re-empower and inform communities otherwise excluded from policy formation (25).

Future guidance to clinicians should emphasize impeccable follow-up of all patients with impetigo in order to corroborate clinical resolution of infection and continue to reinforce the importance of antibiotic adherence. In a population that is highly mobile between primary healthcare providers, effective strategies will need to be developed in partnership with Aboriginal communities themselves. Finally, the preconditions of access to clean water, sanitation, and other enablers for personal hygiene essential to reduce antimicrobial resistance have been powerfully argued (2). For example, the first of six national strategies recommended by the Global Antibiotic Resistance Partnership is to reduce the need for antibiotics by improving access to clean water and functional sewerage systems, and ensuring a safe and healthful food supply (1). In remote Aboriginal communities in the Kimberley, basic sanitation, water, and housing stock including health hardware in the home have been repeatedly shown to be substandard (26, 27). As does the World Health Organization (28), we are convinced that these environmental aspects should also be a focus for local AMS Committees. In the Kimberley, impetigo and other skin infections are directly attributable to the environment (29). Primary healthcare services could address not only clinical prescribing for impetigo but also local partnerships with environmental health services to address environmental determinants and deliver sustained environmental health promotion. AMS risks make this a pressing priority. Forward-looking AMS Committees should maintain the broadest awareness of the multitude of factors that exacerbate antibiotic resistance (30).

## Author Contributions

SO, JC, and JW: all participated in the literature review; concept and design of the survey process; analysis and interpretation of data; drafting and revising the manuscript; and final approval prior to submission for publication. All authors agree to be accountable for the content of the work.

## Conflict of Interest Statement

The authors declare that the research was conducted in the absence of any commercial or financial relationships that could be construed as a potential conflict of interest.

## Footnotes

^1^http://www.kahpf.org.au/.

^2^https://www.naps.org.au.

^3^https://tgldcdp.tg.org.au/etgcomplete.

^4^http://resources.kamsc.org.au/protocols.html.

^5^http://103.18.109.102/~kamscorg/wp-content/uploads/2015/04/oth-Skin-Infections.pdf.

^6^https://www.health.qld.gov.au/townsville/media-releases/2015/150117-rheumatic-heart-disease.asp.

## Abbreviations

ACCHO, Aboriginal Community Controlled Health Organisation; APSGN, acute post-streptococcal glomerulonephritis; ARF, acute rheumatic fever; AMS, antimicrobial stewardship; ca-MRSA, community-acquired methicillin-resistant *Staphylococcus aureus*; DDDs, daily defined doses; DOT, directly observed therapy; GAS, group A *Streptococcus pyogenes*; GARP, Global Antibiotic Resistance Partnership; KSDL, Kimberley standard drug list; LAB, LA-Bicillin; MRSA, methicillin-resistant *Staphylococcal aureus*; NAPS, National Antimicrobial Prescribing Survey; PK/PD, pharmacokinetic/pharmacodynamic; RANs, remote area nurses; RHD, rheumatic heart disease; SXT, sulfamethoxazole/trimethoprim (“co-trimoxazole”); WACHS, Western Australian Country Health Service.
